# The adenosinergic system is involved in sensitization to morphine withdrawal signs in rats—neurochemical and molecular basis in dopaminergic system

**DOI:** 10.1007/s00213-016-4289-7

**Published:** 2016-04-18

**Authors:** Joanna Listos, Irena Baranowska-Bosiacka, Agnieszka Wąsik, Sylwia Talarek, Maciej Tarnowski, Piotr Listos, Małgorzata Łupina, Lucyna Antkiewicz-Michaluk, Izabela Gutowska, Marta Tkacz, Anna Pilutin, Jolanta Orzelska-Górka, Dariusz Chlubek, Sylwia Fidecka

**Affiliations:** Department of Pharmacology and Pharmacodynamics, Medical University of Lublin, Chodźki 4a St., 20-093 Lublin, Poland; Department of Biochemistry and Medical Chemistry, Pomeranian Medical University, Powstańców Wlkp. 72 Av., 70-111 Szczecin, Poland; Department of Neurochemistry, Institute of Pharmacology, Polish Academy of Sciences, Smętna 12 St., 31-343 Kraków, Poland; Department of Physiology, Pomeranian Medical University, Powstańców Wlkp. 72 Av., 70-111 Szczecin, Poland; Department of Pathological Anatomy, Faculty of Veterinary Medicine, University of Life Sciences, Głęboka 30 St., 20-612 Lublin, Poland; Department of Biochemistry and Human Nutrition, Pomeranian Medical University, Broniewskiego 24 St., 71-460 Szczecin, Poland; Department of Histology and Embryology, Pomeranian Medical University, Powstańców Wlkp. 72 Av., 70-111 Szczecin, Poland

**Keywords:** Morphine withdrawal signs, Naloxone, Dependence, Morphine withdrawal signs, Dopamine receptor expression

## Abstract

**Rationale:**

Experimental data informs that not only do the dose and time duration of dependent drugs affect the severity of withdrawal episodes. Previous withdrawal experiences may intensify this process, which is referred as sensitization to withdrawal signs. Adenosine and dopamine (DA) receptors may be involved in this sensitization.

**Objectives:**

Rats were continuously and sporadically treated with increasing doses of morphine for 8 days. In rats, sporadically treated with morphine, morphine administration was modified by adding three morphine-free periods. Adenosine agonists were given during each of the morphine-free periods (six injections in total). On the 9th day, morphine was injected. One hour later, naloxone was administered to induce morphine withdrawal signs. Then, the animals were placed into cylinders and the number of jumpings was recorded. Next, the rats were decapitated and brain and brain structures (striatum, hippocampus, and prefrontal cortex) were dissected for neurochemical, molecular, and immunohistochemical experiments within DAergic pathways.

**Results:**

We demonstrated that previous experiences of opioid withdrawal intensified subsequent withdrawal signs. Adenosine ligands attenuated the sensitization to withdrawal signs. In a neurochemical study, the release of DA and its metabolites was impaired in all structures. Significant alterations were also observed in mRNA and protein expression of DA receptors.

**Conclusions:**

Results demonstrate that intermittent treatment with morphine induces alterations in the DAergic system which may be responsible for sensitization to morphine withdrawal signs. Although adenosine ligands attenuate this type of sensitization, they are not able to fully restore the physiological brain status.

## Introduction

Chronic treatment with morphine and other opioids produces addiction, while cessation of morphine administration induces withdrawal symptoms (Chartoff et al. 2006; Georges et al. 1999; Kaplan and Coyle 1998). In most experimental procedures, morphine withdrawal signs are induced by administration of increasing morphine doses for several consecutive days (Balter and Dykstra 2012; Hao et al. 2011; Trang et al. 2003). However, in cases of addicted patients, chronic treatment with different abusing substances is often interspersed with drug-free periods, e.g., the periods of sleep or impossibility of taking the drug. Moreover, according to experimental data, not only do the dose and time duration of the treatment with addictive drugs affect the severity of withdrawal episode but previous withdrawal experiences may also intensify this process, what is referred to as sensitization to withdrawal signs (Mizutani et al. 2005; Ward and Stephens 1998). For example, certain sensitization to withdrawal signs was observed after repeated ethanol withdrawal (increase in the number of seizure episodes) (Becker et al. 1997; Veatch and Becker 2002). Similarly, some increase in the intensity of withdrawal symptoms was observed after repeated morphine withdrawals in guinea pigs (Mizutani et al. 2005) and after repeated heroine withdrawals in rats (Schulteis et al. 1997). Subsequent studies with diazepam, another addictive compound, also confirmed the sensitization development to withdrawal signs (Listos et al. 2008a; Ward and Stephens 1998). However, the exact mechanisms, underlying this sensitization process, are still rather poorly identified.

It is well known that the dopamine (DA) and DAergic mesocortical limbic system plays an important role in morphine effects, with increased DA levels in the nucleus accumbens after acute morphine doses (Di Chiara 2000). Other brain structures, such as the ventral tegmental area, the ventral striatum, the amygdala nuclei, and the prefrontal cortex nuclei, are also involved in the activity of opioids (Koob et al. 2004). A cessation of chronic treatment with morphine induces opposite effects, such as decreased DA neurotransmission in the nucleus accumbens, which underlies anhedonia and dysphoria during withdrawal period (Acquas et al. 1991; Koob et al. 1989). DA exerts its effects on five DA receptors, out of which D1 and D2 receptors are most recognized and their role in the addiction process is undisputed (Goodman 2008). However, alterations in sensitization to morphine withdrawal signs, observed in the DAergic system, have not been studied.

Adenosine, a potent inhibitory neuromodulator in the central nervous system, acts via four most recognized adenosine receptor subtypes: A1, A2A, A2B, and A3. A1 receptors are highly expressed in different brain areas, such as the cortex, the cerebellum, the hippocampus, and the dorsal horn of the spinal cord. The distribution of A2A receptors is more limited, mainly in the striato-pallidal GABAergic neurons and the olfactory bulb. In other brain areas, their expression levels are much lower (Ferré et al. 1997). It has repeatedly been shown that adenosine is involved in the function of opioid system. It is known that extracellular adenosine levels are increased after exposure to morphine or to the other addictive drugs (Baldo et al. 1999), while adenosine may be responsible for modification of addictive behaviors in animals. In experimental studies, the number of A1 adenosine receptors in the brain presented an increase, contrary to decreased A2A receptors in the striatum, demonstrated in morphine-dependent rats (Ahlijanian and Takemori 1986; Kaplan et al. 1994). Furthermore, a close functional in vitro interaction was also described between adenosine A1, A2A receptors and morphine withdrawal, see Capasso and Gallo (2009). Some involvement of adenosine ligands in the effect of chronic opiate treatment was also demonstrated in behavioral experiments. For example, adenosine agonists were able to reduce opiate withdrawal signs in mice (Kaplan and Sears 1996), as well as the development of morphine sensitization was inhibited by adenosine receptor antagonists in a C57BL/6 mouse (Weisberg and Kaplan 1999). We previously indicated that stimulation of adenosine A2A receptors attenuated the development of hypersensitivity to acute morphine doses during its withdrawal (Listos et al. 2008b). We also showed that adenosine agonists significantly attenuated morphine-induced sensitization to the locomotor activity of mice, which was reflected by morphine seeking behavior (Listos et al. 2011).

Taken together, all the above-mentioned data inspired us to find out if sporadic morphine treatment, intermittent with repeated withdrawal periods, produced sensitization to naloxone-induced withdrawal signs in rats. Out of various withdrawal signs—jumpings in that case, as the most intense opioid withdrawal signs, were selected for evaluation of that sensitization type. Subsequently, the involvement of adenosine A1 and A2A receptors in that sensitization mode was also studied. Additionally, in order to elucidate the mechanisms of sensitization to morphine withdrawal signs, some series of neurochemical and molecular experiments were also performed. In a neurochemical study, using the high-performance liquid chromatography with electrochemical detection (HPLC-ED) method, the concentration levels of DA and its metabolites were assessed in those brain structures which are strongly involved in addiction process, such as the striatum, the hippocampus, and the prefrontal cortex (Koob et al. 2004). The DA is catabolized by two ways: via monoaminooxidase B (MAO-dependent oxidative pathway) synthesized intraneuronal metabolite—3,4-dihydroxyphenylacetic acid (DOPAC); via catechol-O-methyltransferase (COMT-dependent o-methylation pathway) synthesized the extraneuronal metabolite, 3-methoxytyramine (3-MT). The final metabolite of DA is homovanillic acid (HVA). In molecular experiments, the mRNA and protein expression of DA receptors D1 and D2 was investigated in the same brain areas. Finally, immunohistochemical changes in the expression of DA receptors were examined in the hippocampus. The striatum is a brain structure which receives the inputs from the basal ganglia, the cortex, the amygdala, and from other structures. It is strongly involved in various aspects of addiction status (Delgado et al. 2003; Everitt and Robbins 2005; Tanaka et al. 2004). Striatum contains almost exclusively DA neurons. Contrary to this, the hippocampus and cortex contain far fewer DA projections; therefore, it is worth studying the alterations within all these structures. The distribution of D1 and D2 receptors in these structures is also diversified. The hippocampus is a structure, mainly involved in learning and remembering processes. Although it is not directly involved in the activity of abused drugs, it was indicated that the hippocampus could mediate the rewarding effects of morphine by D1 and D2 receptors in the CA1 region (Esmaeili et al. 2012). The prefrontal cortex is an area which is implicated in cognitive behaviors, personality expression, and in moderating social behaviors. It sends the neurons that innervate striatal and midbrain cell regions and it may thereby be involved in the state of dependence. An identification of molecular, neurochemical, and immunohistochemical changes within those structures comprehensively explain the mechanisms, underlying the behavioral differences between the rats, chronically and sporadically treated with morphine, and those which were treated with adenosine ligands. The presented experiments may play a key role in further investigations on morphine effects and in searching for new therapeutic strategies for morphine-addicted patients.

## Materials and methods

### Animals

The experiments were carried out on male Wistar rats (180–200 g). The animals were kept 8–10 per cage at a room temperature of 22 ± 1 °C on natural day-night cycles (12 h/12 h). Standard food (Murigan pellets, Bacutil, Motycz) and tap water were freely available. All the experiments were performed between 8 a.m. and 3 p.m.

The study was performed according to the National Institute of Health Guidelines for the Care and Use of Laboratory Animals and the European Community Council Directive for Care and Use of Laboratory Animals of 24 November (86/609/EEC) and were approved by Committee on the Ethics of Animal Experiments of the Medical University of Lublin (No 29/2012, No 1/2013).

### Drugs in behavioral experiments

The following drugs were used:

- Morphine hydrochloride (Pharma-Cosmetic, Poland);
- Naloxone hydrochloride (Sigma, St. Louis, MO, USA)—opioid receptor antagonist;
- N^6^-cyclopentyladenosine (CPA)—the selective adenosine A_1_ receptor agonist;
- 2-p-(2-Carboxyethyl) phenethylamino-5′-N-ethylcarboxamidoadenosine hydrochloride (CGS 21680)—the selective adenosine A_2A_ receptor agonist.

All drugs were dissolved in 0.9 % saline and they were given intraperitoneally (ip).

Morphine was used at increasing doses (from 10.0 to 50.0 mg/kg, ip). Naloxone hydrochloride was administered at a dose of 2.0 mg/kg. Both adenosine ligands were given at the dose of 0.1 mg/kg. All drugs were administered in a volume of 10.0 ml/kg. Control animals received the same volume of saline at the respective time before the test.

### Procedure of addiction and behavioral experiments

In order to obtain the state of dependence, the animals were treated with increasing doses of morphine: 10.0, 15.0, 20.0, 25.0, 30.0, 35.0, 40.0, 50.0 mg/kg (for 8 days). Each dose of morphine was given twice a day. We wanted to demonstrate that sensitization to naloxone induced morphine withdrawal signs developed in those rats and that the adenosinergic system was involved in that process; thus, we divided the animals into the following groups: (1) *morphine group*—consecutive morphine administrations, according to the above scheme; (2) *morphine*-*sensitized group*—morphine administration was modified by three morphine-free periods. Thus, the administration of morphine was divided into four 2-day periods (4 × 2), interspersed with 36-h morphine-free periods. During each morphine-free period, animals received two injections of saline; (3) *CPA in morphine-sensitized group*—the animals received morphine, as in the *morphine-sensitized group*, while during each morphine-free period, the rats received two injections of CPA in 12-h intervals; (4) *CGS in sensitized group*—the animals received morphine as in the *morphine sensitization group* and, during each morphine-free period, the rats received two injections of CGS 21680 in 12-h intervals; (5) *saline*—those animals received saline in two patterns: for 9 consecutive days and, analogously to the *morphine*-*sensitized group*, it means, for 12 consecutive days. No behavioral differences were observed between these groups; therefore, the status of *saline group* was assigned to them.

On the last day of the study (on the 9th day in the morphine group and the saline group and on the 12th day in the morphine-sensitized group and the CPA/CGS 21680 in morphine-sensitized group), a subsequent dose of morphine (50.0 mg/kg) was injected. One hour later, naloxone (2.0 mg/kg) was administered to induce morphine withdrawal signs in the rats. The animals were then separately placed in glass cylinders and the number of jumpings was recorded for the period of 30 min.

After the end of behavioral experiments, the rats were killed by decapitation and their brains and brain structures (the striatum, the hippocampus, and the prefrontal cortex) were dissected.

**Scheme 1:**
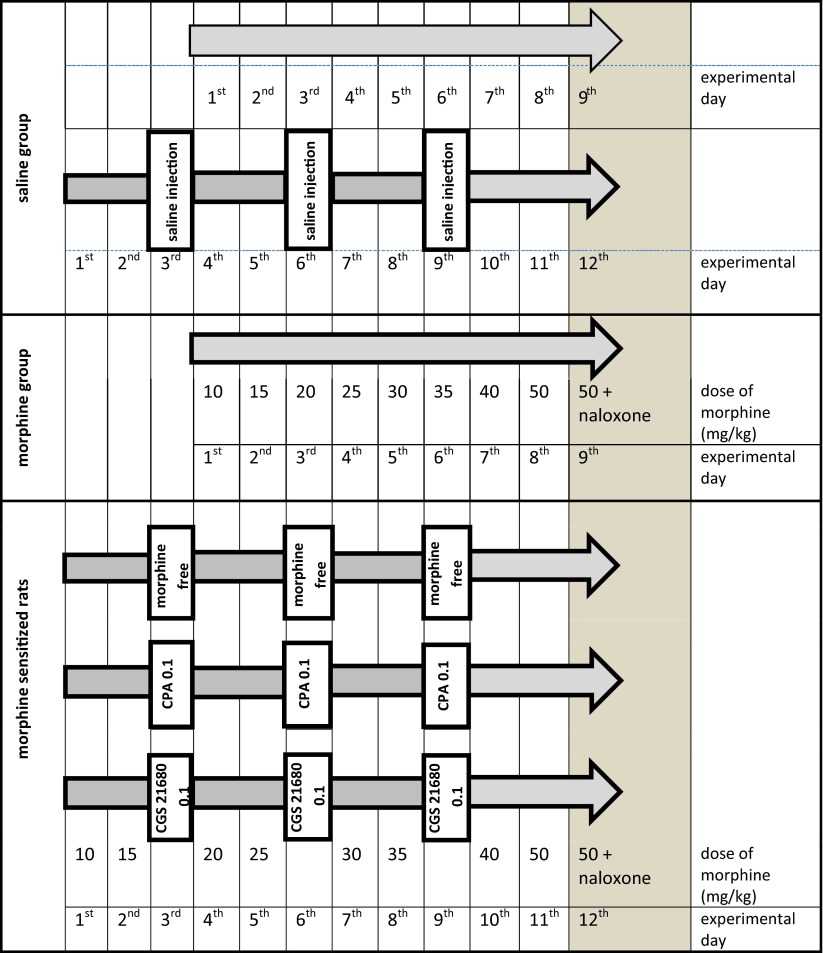
Shows the procedure of administration of morphine in morphine group and morphine and adenosine agonists (CPA and CGS 21680) in morphine-sensitized group

The experimental protocol is graphically depicted in the Scheme 1.

### Neurochemical analysis—ex vivo biochemical studies

After behavioral experiments, the rats were killed by decapitation and their brain structures, including the striatum, the hippocampus, the prefrontal cortex, and the cerebellum, were immediately dissected and the obtained tissues were frozen on solid CO_2_ (−70 °C) and stored until biochemical assay. DA and its metabolites, DOPAC, 3-MT, and the final metabolite, HVA, were assayed by means of HPLC-ED. A HP 1050 chromatograph of Hewlett-Packard, Golden, CO, USA, was equipped with C18 columns. Tissue samples were weighed and homogenized in ice-cold 0.1 M perchloroacetic acid, containing 0.05 mM of ascorbic acid. After centrifugation (10,000×*g*, 5 min), the supernatants were filtered through RC 58 0.2-im cellulose membranes (Bioanalytical Systems, West Lafayette, IN, USA). The mobile phase consisted of 0.05 M citrate-phosphate buffer, pH 3.5, 0.1 mM of EDTA, 1 mM of sodium octyl sulfonate, and 3.5 % methanol. The flow rate was maintained at 1 ml/min. DA and its metabolites were quantified by peak height comparisons with standards run on the day of analysis.

### Molecular analysis

#### The analysis of gene expression by real-time quantitative reverse transcription PCR (RQ-PCR) in the striatum, the hippocampus, and the prefrontal cortex

Total RNA was extracted from 50 to 100 mg brain samples, using an RNeasy Lipid Tissue Mini Kit (Qiagen). After determination of the quantity and quality of isolated RNA, using a NanoDrop ND-1000 spectrophotometer (NanoDrop Technologies, USA), cDNA was prepared from 1 μg of total cellular RNA in 20 μl of reaction volume, using a First Strand cDNA synthesis kit and oligo-dT primers (Fermentas). A quantitative assessment of mRNA levels was performed by real-time RT-PCR on an ABI 7500Fast instrument with Power SYBR Green PCR Master Mix reagent. The real-time conditions were as follows: 95 °C (15 s), 40 cycles at 95 °C (15 s), and 60 °C (1 min). According to melting point analysis, only one PCR product was amplified under those conditions. Each sample was analyzed in two technical replicates and the mean Ct values were used for further analysis. The relative quantity of target, normalized to the endogenous control Gapdh gene and relative to a calibrator, is expressed as 2-∆∆Ct (−fold difference), where Ct is the threshold cycle, ∆Ct = (Ct of target genes) − (Ct of endogenous control gene, β-2 microglobulin), and ∆∆Ct = (∆Ct of samples for the target gene) − (∆Ct of calibrator for the target gene). The following primer pairs were used:

- D1R F: CGC GTA GAC TCT GAG ATT CTG AAT T, D1R R: GAG TTA AGG AGC CAC CAC ATC AGT; D2R F: TGA CAG TCC TGC CAA ACC AGA GAA, D2R R: TGG GCA TGG TCT GGA TCT CAA AGA; Gapdh F: ATG ACT CTA CCC ACG GCA AG, Gapdh R: CTG GAA GAT GGT GAT GGG TT

#### The analysis of protein expression by the western blotting method in the striatum, the hippocampus, and the prefrontal cortex

A RIPA buffer (pH 7.4), containing 20 mM of Tris, 0.25 mM of NaCl, 11 mM of EDTA, 0.5 % NP-40, 50 mM of sodium fluoride, and protease, phosphatase inhibitors (Sigma, Poland) was used for homogenization of the brain samples (Yant et al. 2003). The total protein concentrations were determined, using the Bradford Protein Assay (Sigma, Poland) and the homogenates were subjected to SDS-polyacrylamide gel electrophoresis and assessed for the expression of D1 and D2 receptors. Briefly, the extracted proteins (20 μg/well) were separated on 12 % gel (SDS-PAGE), using a Mini Protean Tetra Cell System (Bio-Rad, Poland). The fractionated proteins were transferred onto a 0.2-μm PVDF membrane (Bio-Rad, Poland), next membranes were blocked with 3 % bovine serum albumin (BSA) solution in buffer for 1 h at room temperature. The brain protein expression was identified, using antibody against D1-rabbit polyclonal (SantaCruz Biotechnology, cat no sc-14001), D2-mouse monoclonal (SantaCruz Biotechnology, cat no sc-5303), GFAP-mouse monoclonal (SantaCruz Biotechnology, cat no sc-33673), and appropriate sAb bovine anti-rabbit/anti-mouse IgG HRP (Santa Cruz Biotech, USA);

The membranes were processed with an ECL Advance Western Blotting Detection Kit (Amersham Life Sciences, UK) and, subsequently, bands were visualized, using the Gel DOC-It Imaging system.

#### Immunohistochemical analysis of D1 and D2 receptors in the hippocampus

The dissected brains were fixed in Carnoy liquid (1 h) and then washed with absolute ethanol (three times within 3 h), absolute ethanol with xylene (1:1) (twice within 1 h), and xylene (3 times within 20 min), and then, after 3-h saturation of tissues with liquid paraffin, the samples were embedded in paraffin blocks. Using a microtome (MICROM HM340E), 3–5 μm serial sections were obtained and placed on silane histological slides (3-aminopropyl-trietoxy-silane, Thermo Scientific, UK). The preparations were deparaffinized in xylene and ethanol with decreasing concentration and then used for further histological staining. In order to expose epitopes, the deparaffinized sections were twice boiled in a microwave oven (700 W, 4 and 3 min) in 10 nM citrate buffer (pH 6.0). Once cooled and washed with phosphate-buffered saline (PBS), the preparations were incubated for 60 min at room temperature with a primary antibody against D1, D2, like used for western blotting analysis, in dilution 1:1000.

In order to visualize the antigen-antibody complex for rabbit antibodies under light microscopy (Axioskop Zeiss, Germany), we used the DAKO LSAB+System-HRP (DakoCytomation, UK), based on the reaction avidin-biotin-horseradish peroxidase with diaminobenzidine (DAB) as chromogen. The result of that reaction was visualized, using diaminobenzidine reaction (DAB, Sigma-Aldrich, Poland).

### Statistical analysis

**Fig. 1:**
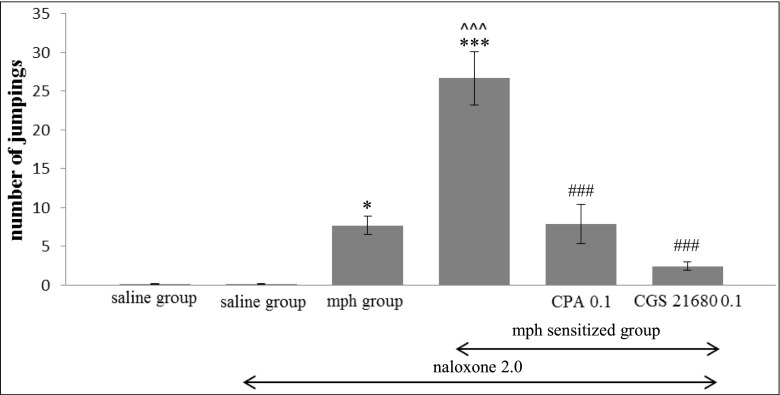
Effects of continuous and sporadic treatment with increasing doses of morphine on the intensity of naloxone-induced morphine (mph) withdrawal signs (jumpings). The role of adenosine A1 and A2A agonists. Rats were treated in two schemes: for 8 continuous days (*mph group*) and for four 2-day periods (4 × 2) interspersed with morphine-free period days (*mph-sensitized group*). Adenosine agonists (CPA—0.1 mg/kg, ip and CGS 21680—0.1 mg/kg, ip) were administered during the morphine-free periods. Naloxone (2.0 mg/kg, ip) was administered for induction of morphine withdrawal signs. *p < 0.05, ***p < 0.001 vs saline group, ^^^*p* < 0.001 vs mph group, ^###^ *p* < 0.001 vs mph-sensitized group (Tukey’s test)

The behavioral results (see Fig. 1) and the neurochemical results (see the Table 1) are presented as the mean values ± S.E.M. Those results were evaluated by the analysis of variance (one-way ANOVA), using the Graph-Pad Prism Software package (version 5.04). The *post*-*hoc* comparisons were carried out, using the Tukey’s test. The total catabolic rate of DA was assessed as the ratio of the concentration of each metabolite (DOPAC, 3-MT, HVA) to that of DA and was expressed as the catabolic rate index: [metabolite] / [DA] × 100. A probability value at *p* < 0.05 was considered as statistically significant. Each group of the animals consisted of 8–10 rats. The results of morphine group and morphine-sensitized group were compared to saline group, while the results of CPA/CGS 21680 in morphine-sensitized group were compared to morphine-sensitized group.

**Table 1:** Presents the concentrations of the dopamine and its metabolites and metabolite/dopamine ratios in rat brain structures

	Treatment	DA (ng/g tissue)	DOPAC (ng/g tissue)	3-MT (ng/g tissue)	HVA (ng/g tissue)	DOPAC/DA (ng/g tissue)	3-MT/DA (ng/g tissue)	HVA/DA (ng/g tissue)
Striatum	Saline	10,524 ± 278.7	1679 ± 87.45	751.3 ± 30.61	1049 ± 71.87	15.95 ± 1.42	7.13 ± 0.34	9.96 ± 0,63
	Saline + naloxone	10,291 ± 851.6	1425 ± 112.1	840.8 ± 126.2	814.2 ± 25.16	13.84 ± 0.52	8.17 ± 2.51	7.91 ± 0.44
	Morphine group + naloxone	7733 ± 604.1 **	1594 ± 114.5	583 ± 52.01	1360 ± 127.7	20.61 ± 1.03	7,53 ± 0.2	17.58 ± 1.09 *
	Morphine-sensitized group + naloxone	9496 ± 340.3	1765 ± 90.25	635.5 ± 49.82	1124 ± 99.23	18.58 ± 0.97	6.69 ± 0.56	11.83 ± 0.73
	CPA in morphine-sensitized group + naloxone	11,177 ± 280.9	1939 ± 111.3	551 ± 22.78	1327 ± 116	17.34 ± 1.28	4.92 ± 0.22	11.87 ± 0.91
	CGS 21680 in morphine-sensitized group + naloxone	10,774 ± 361.1	1750 ± 67.27	558 ± 21.92	1284 ± 63.5	16.24 ± 0.7	5.17 ± 0.38	11.91 ± 0.41
Hippocampus	Saline	13.4 ± 1.046	8.8 ± 1.583	6.167 ± 0.8424	10.25 ± 1.449	65.67 ± 6.3	46.02 ± 18.43	76,49 ± 11.59
	Saline + naloxone	8.8 ± 1.831	5.667 ± 1.729	4.167 ± 0.3658	6.5 ± 0.6268	64.39 ± 2.2	47.35 ± 8.24	73,86 ± 10.72
	Morphine group + naloxone	17.4 ± 2.864	8.4 ± 1.668	36.4 ± 4.879 ***	11.4 ± 1.809	48.27 ± 10.4	209.19 ± 62.05 ***	65.51 ± 11.51
	Morphine-sensitized group + naloxone	7.2 ± 0.6464 *	3.75 ± 1.146 *	17.6 ± 1.796 **	6.6 ± 0.4989 *	52.08 ± 11.51	244.44 ± 33.07 ***	91,66 ± 19.08
	CPA in morphine-sensitized group + naloxone	7.6 ± 1.5	2 ± 0.365	45 ± 6.501 ###	4.6 ± 0.858	26.31 ± 4.9	592.1 ± 85.73 ###	60.52 ± 20.15
	CGS 21680 in morphine-sensitized group + naloxone	6.4 ± 0.498	2.5 ± 0.379	33 ± 2.921 ##	6.333 ± 0.643	39.06 ± 11.18	515.62 ± 21.27 ###	98.9 ± 14.93
Prefrontal cortex	Saline	48.8 ± 2.816	22.25 ± 1.719	26.6 ± 2.746	39.75 ± 3.976	45.59 ± 6.65	54.5 ± 13.71	81.45 ± 8.62
	Saline + naloxone	53.6 ± 4.287	21.17 ± 0.9115	17.25 ± 0.674	23 ± 1.477	39.49 ± 6.72	32.18 ± 6.38	42.91 ± 5.57
	Morphine group + naloxone	66.6 ± 5.319 *	20.4 ± 2.05	33 ± 5.042	24.8 ± 5.339	30.63 ± 7.39	49.54 ± 9.58	37.23 ± 4.79 *
	Morphine-sensitized group + naloxone	73.2 ± 4.519 ***	37.6 ± 2.473 ***	48.5 ± 6.658 ***	74.7 ± 4.193 ***	51.36 ± 3.72	66.25 ± 20.99	102.04 ± 4.84 *
	CPA in morphine-sensitized group + naloxone	65 ± 1.753	39.17 ± 3.975	81.6 ± 11.61 ##	57 ± 6.594	60.26 ± 7.97	125.53 ± 25.49 #	87.69 ± 8.17
	CGS 21680 in morphine-sensitized group + naloxone	56 ± 2.712	37.67 ± 2.193	53.2 ± 5.131	55.67 ± 1.789	67.26 ± 9.82	95.0 ± 13.52	99.41 ± 4.46

*DA* dopamine, *DOPAC* 3,4-dihydroxyphenylacetic acid, *3*-*MT* 3-methoxytyramine; *HVA* homovanillic acid

*p < 0.05; **p < 0.01;***p < 0.001 (vs saline group); ^#^ *p* < 0.05; ^##^ *p* < 0.01; ^###^ *p* < 0.001 (vs morphine-sensitized + naloxone group) (Tukey’s test)

The molecular results were analyzed, using the Statistica 10.0 Software package. The arithmetical mean value ± SD was calculated for each studied parameter. The distribution of results for individual variables was obtained by the Shapiro-Wilk *W* test. As most of the real-time distributions deviated from the normal distribution, non-parametric tests were used for further analyses. In order to assess the differences between the studied groups, the non-parametric Mann–Whitney *U* test was used. The comparisons between groups were performed analogically to behavioral and neurochemical experiments. A probability value at *p* < 0.05 was considered as statistically significant.

## Results

### Behavioral experiments

#### Effects of continuous and sporadic morphine treatment on the intensity of naloxone-induced morphine withdrawal signs (jumpings). The role of adenosine A1 and A2A agonists

As the one-way analysis confirmed, significant changes were observed in the intensity of withdrawal signs in those rats that had chronically been treated with morphine and adenosine ligands (*F*_4,71_ = 21.18, *p* < 0.0001). The number of jumpings increased in the morphine group (*p* < 0.05) when compared with the *s*aline group. Those episodes were significantly intensified in the morphine-sensitized rats, in comparison with the saline rats and the morphine group rats (*p* < 0.001). Both CPA and CGS 21680 significantly reduced the number of withdrawal episodes in the morphine-sensitized rats (*p* < 0.001), see Fig. 1.

### Neurochemical analysis

#### Influence of continuous and sporadic treatment with morphine on DA and its metabolite concentration in brain structures during withdrawal periods induced by naloxone. The role of adenosine A1 and A2A agonists

In the striatum, the one-way ANOVA analysis revealed significant changes in DA concentration (*F*_5,61_ = 4.835, *p* = 0.001) and HVA/DA ratio (*F*_5,31_ = 3.523, *p* = 0.0145) during the withdrawal period. The post-hoc test demonstrated a significant decrease (*p* < 0.01) in DA concentration in the morphine group vs the saline group rats. Furthermore, no significant changes were observed in the concentration levels of all DA metabolites, but HVA/DA ratio was significantly elevated in the morphine group (*p* < 0.05). No significant changes were observed after an acute dose of naloxone in the saline-treated rats and in morphine-sensitized group. Neither CPA nor CGS 21680 affected the concentration of DA or its metabolites in the morphine-sensitized group rats, see Table 1.

Following ANOVA, significant changes were observed in the concentrations of DA (*F*_5,61_ = 7.085, *p* < 0.0001) and of its metabolites (DOPAC: *F*_5,59_ = 5.068, *p* < 0.0007; 3-MT: *F*_5,63_ = 25.38, *p* < 0.0001; HVA: *F*_5,62_ = 7.727, *p* < 0.0001; and 3-MT/DA ratio: *F*_5,35_ = 4.024, *p* = 0.0065) in the hippocampus of the studied rats. A post-hoc test indicated that an acute dose of naloxone did not produce any changes in the hippocampal DAergic pathways. The concentration of DA was markedly reduced (*p* < 0.05) in the morphine-sensitized group, but not in the morphine group. The levels of two DA metabolites, such as DOPAC and HVA, were also reduced (*p* < 0.05) in that group. Adenosine agonists did not affect the concentrations of DA, DOPAC, or HVA. Conversely, compared to the saline group, the concentration of 3-MT was markedly increased, both in the morphine group (*p* < 0.001) and in the morphine-sensitized group rats (*p* < 0.01) and 3-MT/DA ratio was also elevated in these groups (*p* < 0.001). Both adenosine ligands, CPA and CGS 21680, significantly increased 3-MT levels (*p* < 0.001 and *p* < 0.01, respectively) and 3-MT/DA ratio (*p* < 0.001) in comparison with the morphine-sensitized rats, see Table 1.

In the prefrontal cortex, one-way ANOVA showed significant changes in the concentrations of DA (*F*_5,55_ = 6.807, *p* = 0.0003) and its metabolites (DOPAC: *F*_5,65_ = 9.093, *p* = 0.0001; 3-MT: *F*_5,61_ = 13.09, *p* < 0.0001; HVA: *F*_5,62_ = 22.22, *p* < 0.0001; 3-MT/DA ratio: *F*_5,32_ = 5.538, *p* = 0.0012; and HVA/DA ratio: *F*_5,33_ = 7.689, *p* = 0.0001) in the morphine-treated animals. It was demonstrated in the Tukey’s test that an acute dose of naloxone did not induce any significant changes in comparison with the saline animals. The Tukey’s test showed that DA level was increased both in the morphine group (*p* < 0.05) and in the morphine-sensitized group (*p* < 0.001), in comparison with the saline group. Significant increases (*p* < 0.001) in all the metabolite concentrations (DOPAC, 3-MT, HVA) were observed in the morphine-sensitized group, but not in the morphine group, when compared to the saline group rats. The HVA/DA ratio was reduced in the morphine group (*p* < 0.05), and it was elevated (*p* < 0.05) in the morphine-sensitized group. In comparison with the morphine-sensitized group, 3-MT level (*p* < 0.01) and 3-MT/DA ratio (*p* < 0.05) were significantly elevated by CPA, see Table 1.

### Molecular analysis

#### Influence of continuous and sporadic morphine treatment on D1 receptor mRNA and protein expression in rat brain structures during naloxone-induced withdrawal period. The role of adenosine A1 and A2A agonists

**Fig. 2:**
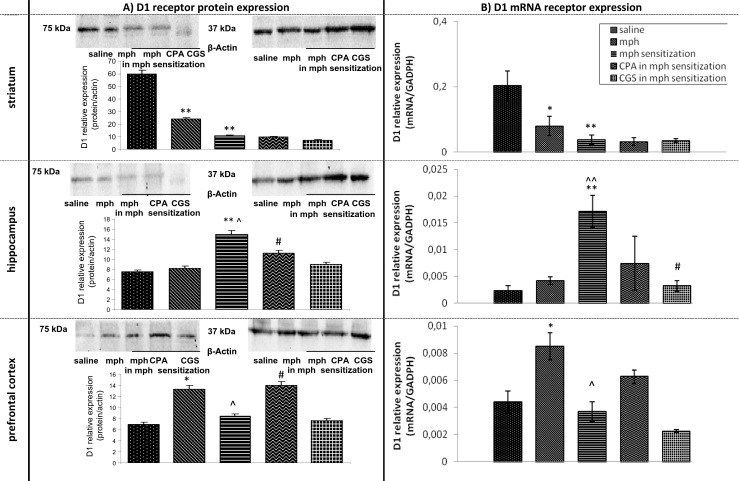
Representative western blots and densitometric analysis of protein (normalized to β-actin) (**a**) and densitometric analysis mRNA levels of D1 receptor (**b**) in brain of continuously and sporadically treated with morphine (mph) rats. The role of adenosine A1 and A2A agonists. The results are expressed as means ± SD from different areas of three to four rat brains. *p < 0.05, **p < 0.01 vs saline group; ^^^ *p* < 0.05, ^^^^ *p* < 0.01 vs mph group; ^#^ *p* < 0.05 vs mph-sensitized group (Mann–Whitney test)

Regarding the striatum, the expression of mRNA (*p* < 0.01) and protein (*p* < 0.05) D1 receptor demonstrated a statistically significant decrease in the morphine group in comparison with the corresponding values in the saline group. A significant down-regulation of mRNA (*p* < 0.01) and protein (*p* < 0.01) of D1 receptor expression was also observed in morphine-sensitized group in comparison with saline group. No statistically significant changes were observed in D1 receptor expression between the morphine group and the morphine-sensitized group in that brain area. Neither CPA nor CGS 21680 affected D1 receptor expression in the morphine-sensitized group rats, see Fig. 2.

In the hippocampus, mRNA and protein expression of D1 receptor were significantly higher (both *p* < 0.01) in the morphine-sensitized group, in comparison to the saline group. No alterations were observed in the morphine group rats vs the saline group rats. The mRNA and protein expression of D1 receptors were significantly up-regulated in the morphine-sensitized group vs the morphine group (*p* < 0.01 and *p* < 0.05, respectively). CGS 21680—an adenosine agonist—down-regulated mRNA of D1 receptor (*p* < 0.05) and CPA down-regulated protein expression of D1 receptors (*p* < 0.05) in comparison with the morphine-sensitized rats, see Fig. 2.

In the prefrontal cortex, increased D1 receptor mRNA (*p* < 0.05) and protein (*p* < 0.05) expression levels were observed in the morphine group vs the saline group, while no significant alterations were found in the morphine-sensitized group, compared to the saline group. D1 receptor expression was significantly down-regulated in the morphine-sensitized group vs morphine group (*p* < 0.05). CPA up-regulated D1 receptor protein expression (*p* < 0.05), in comparison with the morphine-sensitized group, see Fig. 2.

An acute dose of naloxone did not induce any changes in D1 receptor mRNA and protein expression levels in any of the studied parts of the brain, see Fig. 2.

#### Influence of continuous and sporadic morphine treatment on D2 receptor mRNA and protein expression in rat brain structures during naloxone-induced withdrawal period. The role of adenosine A1 and A2A agonists

**Fig. 3:**
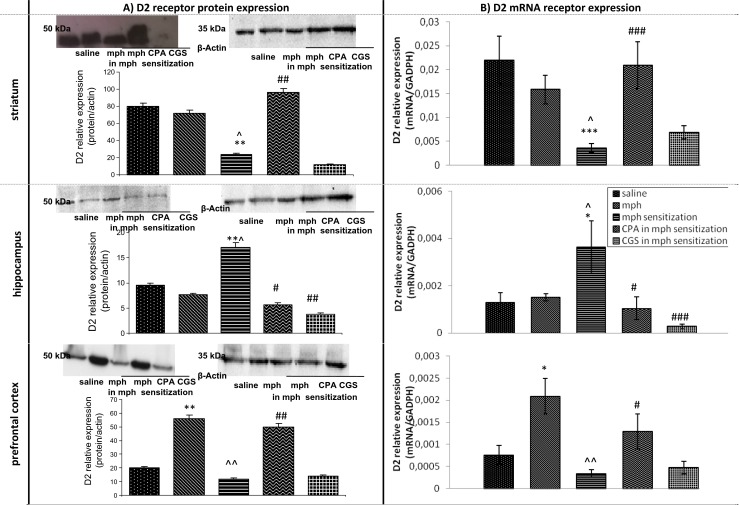
Representative western blots and densitometric analysis of protein (normalized to β-actin) (**a**) and densitometric analysis mRNA levels of D2 receptor (**b**) in brain of continuously and sporadically treated with morphine (mph) rats. The role of adenosine A1 and A2A agonists. The results are expressed as means ± SD from different areas of three to four rat brains. *p < 0.05, **p < 0.01, ***p < 0.001 vs saline group; ^^^ *p* < 0.05, ^^^^ *p* < 0.01 vs mph group; ^#^ *p* < 0.05, ^##^ *p* < 0.01, ^###^ *p* < 0.001 vs mph-sensitized group (Mann–Whitney test)

In the striatum, the *U*-Mann test did not show any significant changes in D2 receptor mRNA or protein expression levels in the morphine group vs the saline group; however, the mRNA and protein expression levels in the morphine-sensitized group were significantly down-regulated (*p* < 0.01 and *p* < 0.001) vs the saline group. D2 receptor expression in the morphine-sensitized group was significantly down-regulated vs the morphine group (*p* < 0.05), see Fig. 3.

CPA significantly up-regulated mRNA (*p* < 0.001) and protein (*p* < 0.01) expression of D2 receptors in comparison with the morphine-sensitized group, see Fig. 3.

In the hippocampus, there were no significant alterations in mRNA nor in protein expression of D2 receptors between the morphine group and the saline group, while D2 receptor expression was significantly increased in the morphine-sensitized group (*p* < 0.05 and *p* < 0.01 appropriately), compared to the saline group. Significant differences were observed between the morphine group and the morphine-sensitized group (*p* < 0.05). Both CPA and CGS 21680 significantly decreased mRNA (*p* < 0.05 and *p* < 0.001, appropriately) and protein (*p* < 0.05 and *p* < 0.01 appropriately) expression of D2 receptors, in comparison with the morphine-sensitized group, see Fig. 3.

In the prefrontal cortex, the expression of mRNA and protein of D2 receptors was significantly up-regulated in the morphine group vs the saline group (*p* < 0.05, *p* < 0.01, respectively), while there were not significant alterations between the morphine-sensitized group and the saline group. D2 receptor expression was significantly down-regulated in the morphine-sensitized group, compared to the morphine group (*p* < 0.01). It was also demonstrated that adenosine agonist, CPA, up-regulated mRNA (*p* < 0.05) and protein (*p* < 0.01) expression, in comparison with the morphine-sensitized group. CGS 21680 did not induce any alterations in expression of D2 receptors in this brain area. The acute dose of naloxone did not induce any changes in D2 receptor expression in any of the studied brain areas, see Fig. 3.

### Immunohistochemical experiments

#### Influence of continuous and sporadic morphine treatment on immunohistochemical changes in D1 receptor expression levels in the rat hippocampus during naloxone-induced withdrawal period. The role of adenosine A1 and A2A agonists

**Fig. 4:**
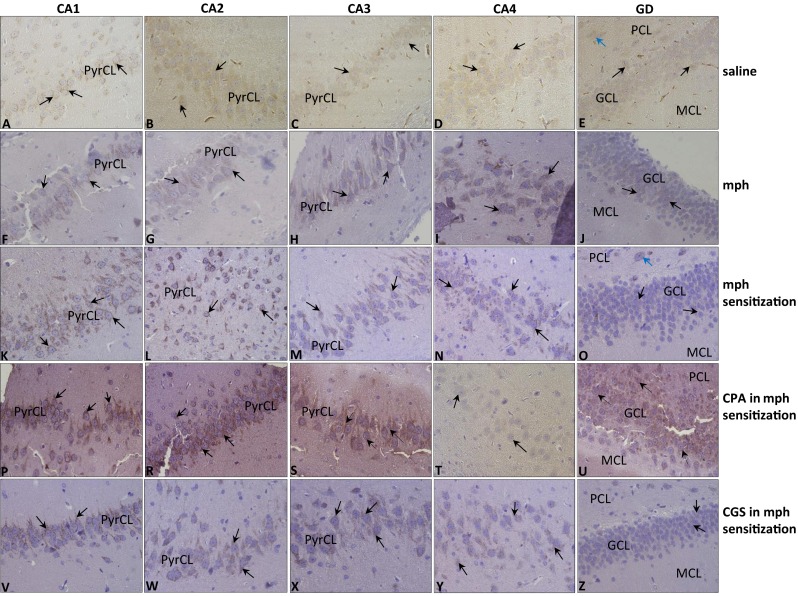
Immunolocalization of D1 receptor in hippocampus of rats during withdrawal period induced by naloxone in: saline group (**A**–**E**); morphine group (*mph*) (**F**–**J**); morphine-sensitized group (*mph sensitization*) (**K**–**O**); CPA in mph-sensitized group (**P**–**U**); and CGS in mph-sensitized group (**V**–**Z**); objective magnification ×40. (CA1–CA4) area of (*CA*) cornu ammonis; (*GD*) gyrus dentate; (*PyrCL*) pyramidal cell layer; (*GCL*) granular cell layer; (*PCL*) polymorphic cell layer; (*MCL*) molecular cell layer

The results of the immunohistochemical reactions showed changes in the expression of D1 receptor in the morphine group (see Fig. 4(F–J)) and in the morphine-sensitized group (see Fig. 4(K–O)), in comparison with the saline group (see Fig. 4(A–E)). The intensity of reaction and the number of immunopositive cells in the morphine group and the morphine-sensitized group were comparable.

In the saline group, D1 receptor positive reactions were observed in cytoplasm and plasmalemma of all the neurons in the *pyramidal cell layer* (PyrCL) of CA1, CA2, CA3 regions (see Fig. 4(A–C); the black arrows) and all the neurons in CA4 region (see Fig. 4(D); the black arrows). Almost all the cells of the *granular cell layer* (GCL) (see Fig. 4(E); the black arrows) and a few larger cells in the *polymorphic cell layer* (PCL) (see Fig. 4(E); the blue arrows) in the *gyrus dentate* exhibited cytoplasmic reaction results.

In the morphine group, the cells of CA1–CA4 regions showed plasmalemmal/cytoplasmic receptor expression (see Fig. 4(F–I); the black arrows), whereas only some of the cells in GCL of gyrus dentate revealed an immunopositive reaction in cytoplasm and plasmalemma (see Fig. 4(J); the black arrows).

Very intensive plasmalemmal/cytoplasmic receptor expression, stronger than in the saline and in the morphine group, was observed in all the neurons of CA1 and CA2 regions of PyrCL in the morphine-sensitized group (see Fig. 4(K, L); the black arrows). Plasmalemmal/cytoplasmic receptor expression, more intensive than in the saline group, but comparable to that in the morphine group, was also shown in the neurons of CA3 regions of PyrCL (see Fig. 4(M); the black arrows) and CA4 (see Fig. 4(N); the black arrows). In the gyrus dentate, plasmalemmal/cytoplasmic immunoexpression of D1 receptor, significantly lower than in *cornu ammonis* was observed in all the cells of GCL (see Fig. 4(O); the black arrows) and in the larger cells in PCL (see Fig. 4(O); the blue arrows). The intensity of reaction in gyrus dentate of the morphine-sensitized group was stronger in comparison to gyrus dentate of the saline group and comparable to that in the morphine group.

Regarding the hippocampus, the number of positive cells and the intensity of reaction in PyrCL of the CA1, CA2, and CA3 region (see Fig. 4(P–S); the black arrows), observed in the CPA in morphine-sensitized group, were high and comparable with the corresponding values in the morphine-sensitized group. In the CA4 region, a lower expression (see Fig. 4(T); the black arrows) was evident, in comparison to the receptor expression in the morphine-sensitized group. An immunoexpression of D1 receptor, significantly stronger than that in the morphine-sensitized group was noticed in GCL of the gyrus dentate (see Fig. 4(U)).

In the CGS in morphine-sensitized group, cytoplasm and plasmalemma of pyramidal cells of CA1, CA2, CA3 and cells of CA4 were immunopositive but the intensity of reaction was comparable to that in the morphine-sensitized group only in CA1 regions (see Fig. 4(V); the black arrows). Immunoexpression in the neurons of CA2, CA3, and CA4 (see Fig. 4(W–Y); the black arrows) was noticeably weaker than that in the morphine-sensitized group. Slightly lower immunoexpression was also observed in the GCL of gyrus dentate (see Fig. 4(Z); the black arrows) in comparison to receptor expression in the morphine-sensitized group.

#### Influence of continuous and sporadic morphine treatment on immunohistochemical changes in D2 receptor expression in the rat hippocampus during naloxone-induced withdrawal period. The role of adenosine A1 and A2A agonists

**Fig. 5:**
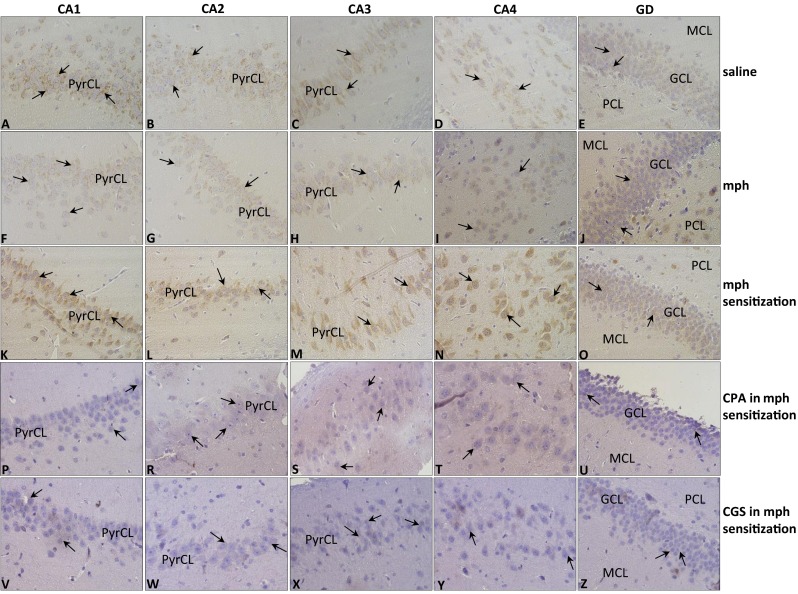
Immunolocalization of D2 receptor in hippocampus of rats during withdrawal period induced by naloxone in: saline group (**A**–**E**); morphine group (*mph*) (F–J); morphine-sensitized group (*mph sensitization*) (**K**–**O**); CPA in mph-sensitized group (**P**–**U**); and CGS in mph-sensitized group (**V**–**Z**); objective magnification ×40. (CA1–CA4) area of (*CA*) cornu ammonis; (*GD*) gyrus dentate; (*PyrCL*) pyramidal cell layer; (*GCL*) granular cell layer; (*PCL*) polymorphic cell layer; (*MCL*) molecular cell layer

In the saline group, explicit D2 receptor immunoexpression was demonstrated in the hippocampus. The pyramidal cells of CA1–CA3 regions (see Fig. 5(A–C); the black arrows) and the neurons of CA4 region (see Fig. 5(D), the black arrows) showed receptor expression in plasmalemma and cytoplasm. In the gyrus dentate, the expression of D2 receptor was also observed in cellular plasmalemma and cytoplasm of the GCL (see Fig. 5(E); the black arrows).

In the morphine group, cytoplasm and plasmalemma of all the neurons in PyrCL of CA1, CA2, CA3 (see Fig. 5(F–H); the black arrows) and the cells of CA4 (see Fig. 5(I); the black arrows) were immunopositive but the intensity of reaction was insensibly weaker than that in the saline group. In the gyrus dentate (see Fig. 5(J); the black arrows), the number of D2 receptor positive cells and the intensity of reaction were comparable with receptor expression in the saline group.

Very intensive D2 receptor expression was indicated in the cytoplasm and plasmalemma of all the neurons of CA1, CA2, and CA3 regions of PyrCL (see Fig. 5(K–M); the black arrows) and in CA4 region (see Fig. 5(N); the black arrows) in the morphine-sensitized rats. The intensity of reaction in all the parts of cornu ammonis was stronger than that in the saline and morphine group. In the gyrus dentate, the plasmalemmal/cytoplasmic immunoexpression of D2 receptor, significantly lower than that in cornu ammonis, was observed in all the cells of the GCL (see Fig. 5(O); the black arrows). The intensity of reaction in gyrus dentate in the morphine-sensitized group was comparable with reaction in gyrus dentate of the saline and the morphine group.

In the hippocampus of the CPA in morphine-sensitized group, D2 receptor expression, significantly lower than that in the morphine-sensitized group, was evident in all the neurons of CA1–CA4 regions (see Fig. 5(P–T); the black arrows). Lower immunoexpression and a few immunonegative cells were also observed in GCL of gyrus dentate (see Fig. 4(U); the black arrows), in comparison to receptor expression in the morphine-sensitized group.

The immunoexpression of D2 receptor within the hippocampus in CGS in the morphine-sensitized group of rats is shown in Fig. 5(V–Z). The neurons of PyrCL in CA1–CA3 regions and neurons of CA4 region showed plasmalemmal/cytoplasmic D2 receptor immunoexpression (see Fig. 5(V–Y); the black arrows), but the number of immunopositive cells was much lower than that in the morphine-sensitized group. Significantly lower immunoexpression of D2 receptor was noticed in the GCL of gyrus dentate (see Fig. 5(O)). Only a small number of granular cells showed low cytoplasmic reaction results.

## Discussion

In the presented study, the effect of continuous (one withdrawal period) and sporadic (repeated withdrawal period) morphine administration on the intensity of naloxone-induced withdrawal signs was examined. Although, various morphine withdrawal signs were observed in studied animals, such as wet dog shakes, paw tremors, or diarrhea, the number of jumpings was chosen for analysis of behavioral changes, as the most prominent morphine withdrawal symptom. In our study, a continuous treatment with increasing doses of morphine produced the expected state of dependence in rats. The most important effect, observed in the presented experiments, was such that a sporadic treatment with morphine (repeated withdrawal signs) produced a significantly higher number of jumpings in comparison with the signs which were observed in continuously morphine-treated rats. It confirmed the assumption of our investigation that previous experiences of opioid withdrawal intensified subsequent withdrawal signs. This phenomenon is referred to as the sensitization to withdrawal signs. Furthermore, we indicated that the administration of selective adenosine A1 or A2A receptor agonists during particular withdrawal episodes significantly reduced the intensity of subsequent morphine withdrawals. Thus, in the presented study, the important role of the adenosinergic system in suppression of morphine sensitization was evidenced.

In these experiments, naloxone, which is commonly used (Diaz et al. 2003; Done et al. 1992) to assess opioid withdrawal signs, was used in dose of 2.0 mg/kg. That dose was not able to induce any withdrawal signs in the saline and did not influence the concentration of all neurotransmitters’ metabolites, either. Therefore, the obtained results in morphine group and morphine-sensitized group are discussed in comparison with those obtained in saline group.

In order to search for the DAergic mechanisms, underlying the sensitization to morphine withdrawal in examined animals, neurochemical and molecular experiments were carried out in the different brain structures: the striatum, the hippocampus, and the prefrontal cortex.

In neurochemical study, in the striatum, a cessation of morphine administration in the morphine group induced a significant reduction of DA level, in comparison with the saline group. That effect was in accordance with other reports, describing studies on the neurochemical effect of naloxone-precipitated withdrawal period (Diaz et al. 2003; Tokuyama et al. 1996). In morphine group, there was also a trend for increased level of final metabolite—HVA and a statistically significant increase in HVA/DA ratio. Lower concentration of DA and increase in HVA/DA ratio suggest that chronic administration of morphine may accelerate DA catabolism in the striatum. On the other hand, no alterations were observed in concentration of DA and its metabolites in the morphine-sensitized rats which could be connected with repeated withdrawal-induced adaptive changes within DAergic system. Some experiments document that even single dose of morphine induces long-lasting adaptation in mesolimbic system (Marinho et al. 2015; Valjent et al. 2010; Vanderschuren et al. 2001). Moreover, Nestby et al. (1995) observed long-term desensitization of D2 receptors in the rat striatum after cessation of intermittent administration of morphine. In our molecular study, the decrease in D1, but not D2, receptor expression was observed in the morphine group, while stronger reduction in D1 and also D2 receptor expression was seen in the morphine-sensitized group. Le Marec et al. (2001) also showed in the striatum a higher density of D2 receptor in the mice, chronically treated with morphine, and lower D2 density in the sporadically treated group. Briefly, in the striatum, one episode of morphine withdrawal (morphine group) leads to alterations in the activity of DA neurons. They are associated with the reduction of DA concentration (induced by the intensification of DA catabolism) and the decrease in D1 receptor density. While repeated morphine withdrawals (morphine-sensitized group) induce also some adaptive changes—a strong reduction of D1 and D2 receptors, but dopamine metabolism is not expressed. It seems that these differences might be responsible for the higher expression of morphine withdrawal signs in sensitized rats.

In the hippocampus of studied rats, marked neurochemical differences in DA and its metabolites were observed. There were no changes in the concentration of DA or its metabolites (except for 3-MT) in the morphine group that was conformable with literature data (Diaz et al. 2003) but there was a significant decrease in the concentration of DA, DOPAC and final metabolite—HVA in the morphine-sensitized group. Because HVA/DA ratio was unchanged, it can be concluded that sporadic morphine administration did not influence an enzyme level involved in DA metabolism, and, lower concentration of DOPAC and HVA was caused by reduced DA level in hippocampal neurons. Apart from, in the hippocampus, the concentration of another DA metabolite, 3-MT, and 3-MT/DA ratio were significantly elevated, both in the morphine group and the morphine-sensitized group. The lack of difference between these groups seems to have no significance on the expression of behavioral sensitization in studied rats. This marked increase in 3-MT level may be explained by morphine-evoked induction of some enzymes, taking part in DA metabolism. Similar increase in 3-MT level was observed by other authors (Honkanen et al. 1994; Sotnikova et al. 2010) and it may be an important antioxidant defense mechanisms (Miller et al. 1996). Moreover, in molecular study, in the hippocampus, no alterations were found in mRNA and protein expression levels of D1 and D2 receptors in the morphine group, while in the morphine-sensitized group, the expression of these receptors was significantly higher. We found immunoexpression of D1 and D2 receptors in the cells of CA1–CA4 regions of cornu ammonis and in the GCL of gyrus dentate in the morphine group. In the morphine-sensitized group, more intensive immunoexpression of D1 receptor was indicated, especially in the neurons of CA1 and CA2 regions of PyrCL of cornu ammonis, whereas the intensity of D2 receptor immunoexpression was stronger than that in the saline and in the morphine group in all the parts of cornu ammonis. Thus, it seems that higher expression of D1 and D2 receptors in the hippocampus might have been correlated with the reduction of DA and its metabolites (DOPAC and HVA) concentration in that area and might have been responsible for the expression of behavioral sensitization in studied rats.

In the rat prefrontal cortex, DA level in the morphine group was increased in comparison with those in the saline group, which was in accordance with literature data (Bassareo et al. 1995; Rossetti et al. 1993). In this group there was a tendency toward to reduction level of a final metabolite—HVA and a statistically significant decrease in HVA/DA ratio, indicating that chronic administration of morphine may inhibit DA catabolism in the prefrontal cortex. However, in the morphine-sensitized group, the DA level and the concentration of DA metabolites (DOPAC, 3-MT, and HVA) were markedly potentiated in comparison with the saline group. The HVA/DA ratio was also significantly increased showing that repeated morphine withdrawals may stimulate the enzymes involved in DA metabolism. In the molecular study, we demonstrated higher expression of protein and mRNA of D1 and D2 receptors in the morphine group, and those expressions were not noticed in the morphine-sensitized group. Thus, the lower expression of DA receptors in the prefrontal cortex of sensitized rats was correlated with the high levels of DA and its metabolites in that brain structure.

All above discussed results showed substantial differences between morphine group and morphine-sensitized group in the striatum, the hippocampus and the prefrontal cortex, which supports the significance of these brain areas in the expression of morphine sensitization to withdrawal signs.

Regarding behavioral experiments, we also demonstrated that both selective adenosine receptor agonists (CPA and CGS 21680) were able to attenuate sensitization to morphine withdrawal signs in studied rats. In neurochemical study, following the administration of CPA or CGS 21680 in the striatum, no alterations were observed in the concentration of either DA or its metabolites in comparison with the morphine-sensitized group. In molecular study, in the striatum, both CPA and CGS 21680 did not induce any changes in D1 receptor expression, but CPA produced a significant increase in D2 receptor expression in the CPA in morphine-sensitized rats, in comparison with the morphine-sensitized group. Thus, CPA restored the density of D2 receptor to control level which may be important for lower expression of withdrawal signs in sensitized rats.

In the hippocampus, only 3-MT level was significantly elevated by CPA and CGS 21680 in comparison with the morphine-sensitized group. This increase might have been caused by morphine-evoked interactions between adenosine receptors and other receptors (e.g., DA receptors). Taking into account that 3-MT is involved in antioxidant defense mechanisms (Miller et al. 1996), and adenosine acts as endogenous neuroprotective agent (Melani et al. 2014), it can be supposed that observed in the hippocampus significant increase in 3-MT level induced by CPA and CGS 21680 may be an interesting phenomenon needed further studies. In the hippocampus, both CPA and CGS 21680 significantly decreased the expression of D1 and D2 receptors in the CPA and CGS 21680 in morphine-sensitized group, restoring mainly D1, but also D2, receptor levels, to the control density. It may be supposed that some connections between high 3-MT level and adenosine receptors may be involved in these effects, however it requires further studies. Immunohistochemical analysis of the hippocampus revealed, for the first time, that reduced immunoexpression of D1 receptor after the administration of CGS 21680 was especially related to the neurons of CA2, CA3, and CA4 regions of cornu ammonis, while after the administration of CPA or CGS 21680, a significantly lower immunoexpression of D2 receptor was observed in the neurons of CA1–CA4 regions of cornu ammonis and in the neurons of the GCL of gyrus dentate.

In the prefrontal cortex, only 3-MT level was significantly elevated by CPA in comparison with the morphine-sensitized group. CPA also significantly increased expression of D1 and D2 receptor (the expression of mRNA of D1 receptors was close to statistical significance) reversing the changes induced by sporadic morphine administration. The distribution of A2A receptors in prefrontal cortex is poor (Ferré et al. 1997) and this may be the reason for the lack of effect CGS 21680 in that structure.

Thus, in this part of the study, we demonstrated that both selective adenosine receptor ligands were able to attenuate the morphine sensitization to withdrawal signs by influence on the activity of D1 and D2 receptors in three examined brain areas. It should be noted that both ligands were administered during three withdrawal periods, but not in the experimental day, which indicate that they are able to produced long-term effects on the sensitization to morphine withdrawal signs.

Taken together, in the present manuscript, we demonstrate that intermittent morphine administration intensifies the subsequent morphine withdrawal signs (i.e., sensitization to morphine withdrawal signs) and that selective adenosine receptor agonists are able to attenuate this phenomenon in rats. It seems that this model of the sensitization reflects the schedule of morphine administration in addictive humans, taking into account the impossibility to take a drug. Additionally, we show the significant alterations within DAergic system at the neurochemical and the molecular level which are involved in sensitization to morphine withdrawal. Firstly, we have demonstrated changes which are developed during repeated withdrawals: (1) in the striatum, the repeated withdrawals induce strong reduction of DA receptor density, without effect on DA level; (2) in the hippocampus, the significant attenuation of DA concentration and high expression of DA receptor; (3) in the prefrontal cortex, significant increase in DA level without the effect on DA receptor expression. Secondly; we have shown changes in DAergic system which occur after stimulation of adenosine receptors: (1) stimulation of adenosine receptors by CPA or CGS 21680 does not influence on DA and its metabolites concentration in all studied brain areas; (2) in the striatum, the administration of CPA, but not CGS 21680, restore the density of D2 receptor to control level; (3) in the hippocampus, both adenosine ligands reduce the expression of D1 and D2 receptors recovering their level to control values; (4) in the prefrontal cortex, CPA, but not CGS 21680, significantly increased the expression of D1 and D2 receptor density, reversing the changes induced by sporadic morphine administration.

The present experiments have shown how complex and multi-level changes occur after morphine administration, thus posing a challenge to have the alterations reversed with a perspective to develop an effective therapy for morphine-addicted patients.
